# Cognitions in Sleep: Lucid Dreaming as an Intervention for Nightmares in Patients With Posttraumatic Stress Disorder

**DOI:** 10.3389/fpsyg.2020.01826

**Published:** 2020-08-21

**Authors:** Brigitte Holzinger, Bernd Saletu, Gerhard Klösch

**Affiliations:** ^1^Institute for Consciousness and Dream Research, Vienna, Austria; ^2^Certificate Programme Sleep Coaching, Medical University of Vienna, Vienna, Austria; ^3^Department of Psychiatry, Medical University of Vienna, Vienna, Austria; ^4^Department of Neurology, Medical University of Vienna, Vienna, Austria

**Keywords:** sleep quality, therapy, anxiety, depression, posttraumatic stress disorder, lucid dreaming

## Abstract

About 80% of posttraumatic stress disorder (PTSD) patients suffer from nightmares or dysphoric dreams that cause major distress and impact nighttime or daytime functioning. Lucid dreaming (LD) is a learnable and effective strategy to cope with nightmares and has positive effects on other sleep variables. In LDs, the dreamer is aware of the dreaming state and able to control the dream content. The aim of this study is to evaluate the effectiveness of lucid dreaming therapy (LDT) in patients suffering from PTSD. We suggest that learning a technique that enables the affected subjects to regulate the occurrence and content of nightmares autonomously increases the chance of coping with the complex symptoms of PTSD and can reduce suffering. Sleep quality (PSQI, Pittsburgh Sleep Quality Index), daytime sleepiness (ESS, Epworth Sleepiness Scale), quality of life (MQLI, Multicultural Quality of Life Index), psychological distress (SCL-90-R, Symptom Checklist 90-Revised), distress caused by traumatic events (IE-S, Impact of Events Scale), anxiety (SAS, Self-Rating Anxiety Scale), depression (SDS, Self-Rating Depression Scale), and nightmare severity were assessed in a self-rating questionnaire before and after the intervention. LDT had no effect on the investigated sleep variables. No correlation between reduction of nightmare severity and changes in PTSD-profile (IE-S) was found. Nevertheless, levels of anxiety and depression decreased significantly in the course of therapy. LDT could provide an alternate or complementary treatment option for nightmares in PTSD, specifically for symptoms of anxiety and depression.

## Introduction

Nightmares are vivid dream experiences loaded with anxiety or fear, which typically occur during rapid eye movement (REM) sleep and less commonly during N2 sleep (American Academy of Sleep Medicine, 2014). Common themes include failure and helplessness, physical aggression, accidents, being chased, health-related concerns and death, and interpersonal conflicts (Robert and Zadra, 2014; Schredl and Göritz, 2018). If these dysphoric dreams recur with enough frequency, cause major distress and impact nighttime or daytime functioning, they may be classified as nightmare disorder (American Academy of Sleep Medicine, 2014). According to the International Classification of Sleep Disorders, Third Edition (American Academy of Sleep Medicine, 2014), minimal diagnostic criteria are as follows: (1) the patient suffers from repeated episodes of extended, extremely dysphoric, and well-remembered dreams that usually involve threats to survival, security, or physical integrity; (2) on awakening from the dysphoric dreams, the person rapidly becomes oriented and alert; and (3) the dream experience itself or the sleep disorder resulting from it causes significant distress or impairment in social, occupational, or other important areas of functioning. Thorough diagnosis is necessary to assess potential comorbidities like depression and other psychiatric disorders or contributing factors such as medications, substances, and recent or past stressful life events.

Affecting about 4% of the adult population (Levin and Nielsen, 2007) and up to 20% of children and adolescents (American Academy of Sleep Medicine, 2014), frequent nightmares are quite common and have a big impact on quality of life, daytime sleepiness, fatigue, and anxiety. Nightmare distress can lead to problems at work, social and cognitive impairments and was even associated with a higher suicide risk (Nadorff et al., 2018).

Zadra and Donderi (2000) and Robert and Zadra (2014) explain the formation of nightmares as a combination of a certain affect load and the disposition to experience heightened distress and negative affect. With respect to brain physiology, nightmares are suggested to be a result of heightened amygdala and hippocampus activation and a failure of prefrontal regions to dampen this activation of the limbic system (Levin and Nielsen, 2007). This activity pattern is typical for REM sleep and might reflect visual hallucinations, emotional intensifications, and cognitive abnormalities (Hobson and Pace-Schott, 2002). Whereas REM sleep is characterized by wake-like high-frequency electroencephalographic activity (Siclari et al., 2017), reports of dream experiences are also associated with a decrease in low-frequency EEG in posterior cortical regions in both REM and NREM sleep (Siclari et al., 2017).

Nightmares are more prevalent during periods of stress and can emerge in association with traumatic experiences, as in posttraumatic stress disorder (PTSD; Zak et al., 2019). PTSD is the delayed and protracted reaction to a traumatic event or situation, which is likely to cause pervasive distress in almost anyone (World Health Organization, 1992). About 80% of the general population experiences at least one relevant trauma throughout their lives (NIH, 2012), and lifetime prevalence of PTSD is considered to be 7.4%. The disorder is defined by a tendency to avoid people, places, memories, and other stimuli related to the traumatic event, as well as recurrent distressing memories of the event and alterations in mood and hyperarousal (American Psychiatric Association, 2013). Additionally, about 80% of PTSD patients suffer from nightmares (Morgenthaler et al., 2018) which are often emotionally related to the original trauma (Nadorff et al., 2014). Nightmares related to PTSD are equally likely to arise during N1/N2 and REM sleep (Phelps et al., 2018).

Since nightmares represent a chronic and persistent symptom of PTSD and cause major distress by themselves, various treatment options have been discussed. The American Academy of Sleep Medicine listed six cognitive behavioral therapy (CBT) methods as suitable for the treatment of nightmares: imagery rehearsal therapy (IRT), systematic desensitization, lucid dreaming therapy (LDT), exposure, relaxation, and rescripting therapy (ERRT), sleep dynamic therapy, and self-exposure therapy (Aurora et al., 2010). Although each therapy approaches the treatment for nightmares differently, they all conceptualize nightmares as a learned response that can be modified by specific cognitive and behavioral strategies. Hypnosis has also been found to provide fast and effective help by addressing the underlying issue of nightmares (e.g., Eichelman, 1985). Pharmacological approaches to control nightmares include prazosin and selective serotonin reuptake inhibitors. All non-pharmacological techniques target the nightmare’s content during wakefulness, with the exception of LDT, which enables the dreamer to modify the dream content while dreaming.

### Lucid Dreaming Therapy

Lucid dreaming (LD) is defined as the awareness of being in a dream and the ability to volitionally control its content (Holzinger, 2008). LD is characterized by a recovery of reflective cognitive capabilities associated with the reactivation of frontal and frontolateral brain regions (Dresler et al., 2012).

LDT can be summarized as a cognitive-restructuring method which can be applied in the state of dreaming. By learning this technique, the dreamer becomes aware and able to actively influence the dream’s content. This approach delivers a new access on how to cope with nightmares, as LDT allows to alter the story line of the nightmare during the dream phase. LDT has been shown to be effective in narcoleptic patients suffering from PTSD (for a review, see Schiappa et al., 2018). Harb et al. (2016) investigated the relationship between posttraumatic nightmares and the effects of LD, when included in an IRT. In a group of military veterans, they found that the increase of dream content control, as an aspect of LD, led to a reduction in nightmare distress and consequently seemed to contribute to the therapeutic change with IRT. Zadra (1997) and Spoormaker and van den Bout (2006) found that, compared to baseline and waiting-list conditions, not only did the frequency of nightmares decrease significantly, but the quality of sleep increased and PTSD symptoms were alleviated (Krakow et al., 2001), suggesting that LDT may be a helpful therapy. LD has been investigated in adults (Schredl et al., 2012) but studies that examine nightmares under psychological and physiological aspects at the same time are scarce. This can be explained on the one hand by the high costs of polysomnographic recording and on the other hand through the lack of nightmare exploration in sleep laboratory studies. Several studies have demonstrated the positive effects of LDT on nightmare frequency (Spoormaker and van den Bout, 2006) and improvement of quality of sleep (Holzinger et al., 2015). Unfortunately, it often remains unclear which of several investigated variables caused this effect or if there are interactions between them. For example, some participants reported having fewer nightmares although they never became lucid (Gavie and Revonsuo, 2010). Furthermore, the number of participants is often small and may not be representative due to drop-outs, recruitment practices, or similar. On a side note, caution should be taken when dealing with psychotic patients, since there seems to be a risk of LD empowering deliria and hallucinations (Mota et al., 2016). Aside from these exceptions, LD could provide a useful tool for people affected by nightmares, since it could activate self-responsibility and self-control in a frightening situation. In this respect, it presents an advantage over traditional therapeutic treatments as it can be applied in the situation itself, while the nightmare is happening, and not afterwards (Holzinger, 2014) Furthermore, it is theorized that treating nightmares with LD might lead to more ego strength, disappearance of anxiety and obsessive-compulsive symptoms, and increased confidence, emotional safety and balance (Tholey, 1988). These effects have yet to be fully investigated.

The aim of the present study was to (i) evaluate LDT in patients with PTSD with nightmares and (ii) if LDT leads to a sustainable reduction of nightmares. The secondary goal was to investigate the efficiency and sustainability in the reduction of nightmare frequency.

## Materials and Methods

### Participants and Procedure

The final sample included 31 adults suffering from nightmares from an acute stressful situation according to the Impact of Events Scale-Revised (IES-R; Weiss and Marmar, 1997) and seeking treatment. Participants were recruited across Lower Austria *via* an in-patient treatment center for psychiatric patients located in Ybbs. Interested patients were informed of the study’s purpose and returned written informed consents. Subsequently, subjects were medically examined prior to the study, and substance use was common. However, medical history of the participants and descriptions of which substances were used cannot be given here. Subjects were randomly assigned to LDT (*n* = 20, 10 females) or a credible active comparison condition (*n* = 11, eight females) for the treatment of nightmares.

**Figure 1:**
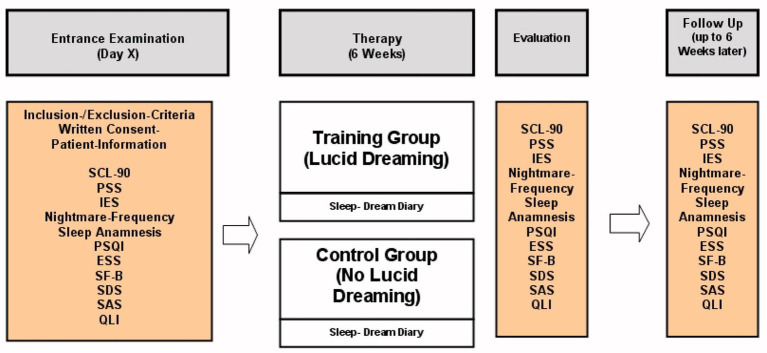
Study design for treatment group and controls.

All 31 subjects kept a sleep diary for the 6 weeks of treatment and completed all of the measures shown in Figure 1. Controls in the active comparison condition did not receive any kind of treatment but were instructed to keep a sleep diary over the course of 6 weeks. Those participants assigned to LDT additionally received one 60-min group session each of the 6 weeks. Follow-up surveys consisted of voluntary returns of the questionnaires and happened 6 weeks after the end of the intervention. Unfortunately, due to comorbid substance use disorder, drop-out rates were high and sample sizes of the LDT group varied between measures (baseline *n* = 9–17, end of therapy *n* = 4–13).

The study was approved by the Ethics Committee of the Medical University of Vienna and Vienna General Hospital (AKH).

### Lucid Dream Therapy Sessions

The 60-min group sessions were held weekly for a total of 6 weeks and took place at the in-patient treatment center in Ybbs, Lower Austria. Sessions were led by two therapists, a doctoral clinical psychologist and the resident clinical psychologist at the center. Due to reasons of documentation and confirmability of LD activity, all sessions were audio-taped. The procedure was applied as follows: the first half-an-hour was designated to the weekly reports of what the participants experienced since the last meeting regarding sleep, dreaming, and LD. Participants shared their dreams in detailed verbal reports. Those dreams were discussed. The group shared their ideas about in which dream scenes the dreamer could have become aware of the dream state and how the dreamer could have changed the dream plot in the dream. The following half-an-hour was used to teach the theoretical background of LD and how to apply self-hypnosis for LD.

### Measurements and Questionnaires

#### Symptom Checklist 90-Revised

The Symptom Checklist 90-Revised (SCL-90-R; Derogatis, 2000; German Version: Franke, 1995) is a 90-item self-report measure to evaluate nine primary symptom dimensions (somatization, obsessive-compulsive, interpersonal sensitivity, depression, anxiety, hostility, phobic anxiety, paranoid ideation, and psychoticism) within the last week. The Global Severity Index indicates the overall psychological distress. Answers are given on a five-point rating scale and completion time is about 12–15 min. The internal consistency coefficient rating ranges from 0.90 for depression and 0.77 for psychoticism. Test-retest reliability has been reported at 0.80–0.90 with a time interval of 1 week.

#### Impact of Events Scale

The IES-R (Weiss and Marmar, 1997) is a 22-item self-report measure that assesses subjective distress caused by traumatic events and asks for the occurrence of symptoms within the past 7 days. Dimensions measured by the IES-R are avoidance, intrusions, and hyperarousal, and items are rated on a five-point scale ranging from 0 (“not at all”) to 4 (“extremely”). Weiss and Marmar (1997) showed that the reliability rates of the subscales are very high with Cronbach’s *α* ranging from 0.79 to 0.92.

#### Pittsburgh Sleep Quality Index

The Pittsburgh Sleep Quality Index (PSQI; Buysse et al., 1989) is a 19-item self-report measure of sleep quality and disturbances during the past month, creating seven component scores for sleep quality, latency, duration, habitual efficiency, disturbance, use of sleep medication, and daytime dysfunction. Each item is weighted on a 0–3 interval scale, creating an overall score from 0 to 21 with scores above 5 indicating poor sleep quality (Buysse et al., 1989). Backhaus et al. (2002) showed that the PSQI has a high test-retest reliability with a coefficient of 0.87 and good validity (sensitivity 98.7 and specificity 84.4).

#### Epworth Sleepiness Scale

The Epworth Sleepiness Scale (ESS; Johns, 1991) is a self-administered questionnaire with eight questions assessing the general level of daytime sleepiness. A total score between 0 and 24 is calculated. The threshold for increased diurnal fatigue is a total score above 10. The test has high internal consistency (Cronbach’s *α* = 0.88–0.74) and reliability (*r* = 0.82).

#### Multicultural Quality of Life Index

The Multicultural Quality of Life Index (MQLI; Mezzich et al., 2011; German version: Katschnig et al., 2003) is a self-rating instrument that measures the subjective quality of life using 10 items that cover concepts from physical well-being to spiritual fulfillment. It is a very economical instrument (mean time of completion under 3 min, Mezzich et al., 2011) with high test-retest reliability (*r* = 0.87) and high internal consistency (Cronbach’s *α* = 0.92).

#### Self-Rating Anxiety Scale

The Self-rating Anxiety Scale (SAS; Zung, 1971) is a 20-item self-report assessment device that measures state and trait anxiety. The total score can range from 20 to 80. Levels from 20 to 44 are considered normal, 45 to 49 indicate mild to moderate anxiety, 60 to 74 indicate severe anxiety, and 75 to 80 indicate extreme anxiety. Ramirez and Lukenbill (2008) found a high internal consistency with a coefficient of 0.80 and convergent validity ranging from 0.21 to 0.60. for the adapted version of the SAS, the SAS-ID (intellectual disabilities).

#### Self-Rating Depression Scale

The Self-rating Depression Scale (SDS; Zung, 1965) consists of 20 statements that have to be rated on a four-point scale (1 = *a little of time* and 4 = *most of the time*). The SDS score is the sum of all responses, and the SDS index is derived by dividing the total score by 80 and multiplying it by 100. A score between 25 and 43 is considered normal, 44 and 49 indicates borderline depression, 50 and 59 mild depression, 60 and 69 moderate to severe depression, and above 70 counts as severe depression (Zung, 1965). The Zung SDS has been shown to have good discriminant validity (Zung, 1965).

#### Perceived Stress Scale

The Perceived Stress Scale (PSS; Cohen et al., 1983) measures the degree to which situations in one´s life are appraised as stressful. The questionnaire comprises ten items, rated on a five-point scale from 0 = *never* to 4 = *very often*.

#### Sleep/Dream Checklist

Participants completed a sleep and dream rating scale daily during intervention phase and reported their dreams during the weekly meetings. To assess nightmare severity, subjects rated their nightmares on a severity scale from 0 = *not severe* to 5 = *very severe*. Additionally, participants were asked how much the nightmares were interfering with their quality of life and with their daytime functioning, both on a scale from 0 = *no interference* to 100 = *very interfering*. Nightmare frequency was also assessed.

The efficiency of LDT was obtained by comparing nightmare frequency at baseline with the nightmare frequency at end of therapy. We expected a decrease of PTSD symptoms due to reduction of nightmare frequency. The extent of improvement of the initial disturbance by nightmares until the end of therapy was assessed by concomitant psychological tests.

### Data Analysis

All analyses were performed after the end of the data collection. For statistical analysis, results at *end of therapy* and *follow-up* were combined due to missing data. Baseline results and *end of therapy/follow-up* results were compared using Mann-Whitney U-test. Wilcoxon tests were used for the longitudinal comparison of baseline and *end of treatment/follow-up* for LDT and control group. The threshold for the rejection of the null hypothesis was set to 0.05. All statistical analyses were performed using the IBM SPSS Statistics for Windows, version 24.0 (IBM, 2016).

## Results

The total sample (*N* = 31) included 18 females and 13 males between 27 and 59 years (*M*_age_ = 41.58 years). The high drop-out rate can be explained due to the high number of participants with a comorbid substance use disorder. This is also the reason for varying sample sizes for each of the measures (Table 1).

**Table 1:** Longitudinal differences in results of lucid dreaming therapy (LDT) group at beginning and end of therapy.

		Baseline		End of therapy		
Measure	*n*	*M*	*SD*	*M*	*SD*	*p*
Pittsburgh Sleep Quality Index (PSQI)	12	11.33	3.33	8.79	3.93	0.149
Epworth Sleepiness Scale (ESS)	4	10.63	4.62	7.00	4.49	0.109
Perceived Stress Scale (PSS)	8	22.00	12.91	25.38	11.81	0.465
Symptom Checklist 90-Revised (SCL-90-R)	12	73.12	9.30	72.83	9.60	0.225
Impact of Events Scale (IE-S)	6	48.31	14.69	39.29	21.48	0.728
Self-rating Anxiety Scale (SAS)	12	46.56	9.59	41.42	9.56	0.012
Self-Rating Depression Scale (SDS)	12	50.93	8.71	45.08	10.92	0.043
Multicultural Quality of Life Index (MQLI)	11	5.07	1.63	5.73	1.85	0.401
Quality of life	12	60.50	30.18	48.63	21.70	0.091
Daily functioning	12	65.00	28.76	63.42	21.38	0.116
Nightmare frequency	13	Several/Month		Several/Month		0.865

### Results at Baseline (Beginning of Therapy)

The LDT group (*n* = 20, 10 females) was 41.58 years on average (*SD* = 8.49). The average body weight was 169.16 lbs (*SD* = 42.66) and average height was 5.70 ft. The average number of nightmares was 3.20 (*SD* = 1.70) per week. Controls (*n* = 11, eight females) were on average 45.38 (*SD* = 10.93) years old, had an average body weight of 156.09 lbs (*SD* = 26.61) and an average height of 5.65 ft.

Means and SDs were calculated for each of the measures; however, sample sizes were different for each one and differ from those found in Table 1: PSQI *n* = 15, ESS *n* = 16, PSS *n* = 9, SCL-90-R *n* = 17, IES *n* = 14, SAS *n* = 16, SDS *n* = 15, MQLI *n* = 14, quality of life *n* = 16, daily functioning *n* = 15, and nightmare frequency *n* = 16. All numbers presented here are of those attending the LDT treatment. The mean score at baseline for sleep quality (PSQI global score) was *M* = 11.80 (*SD* = 3.83), for daytime sleepiness (ESS) it was *M* = 6.50 (*SD* = 3.21), for trauma severity (Items 22–38) it was *M* = 30.40 (*SD* = 9.71), for the Symptom Checklist (SCL-90-R) it was *M* = 73.12 (*SD* = 10.60), for the IE-S (global score) it was *M* = 54.20 (*SD* = 12.07), for anxiety (SAS) it was *M* = 41.00 (*SD* = 8.20), for depression (SDS global score) it was *M* = 51.40 (*SD* = 6.43), for quality of life (MQLI) it was *M* = 5.86 (*SD* = 1.54), for the interference of nightmares with quality of life it was *M* = 60.80 (max. 100), for the interference of nightmares with daily functioning it was *M* = 72.00 (max. 100), and for nightmare frequency it was *M* = 4 (max. 5).

No significant group differences between LDT and controls at baseline except the ESS score (*p* = 0.049; Mann-Whitney U-test) could be found. In summary, both groups (controls and lucid dreamers) demonstrated high levels of daytime sleepiness, with poor sleep quality, severe traumatization and showed symptoms of psychologically distress, anxiety, and depression.

### Results at the End of Therapy

Results for the comparison of baseline and end of therapy scores for those in the LDT condition are presented in Table 1.

In the LDT group, anxiety (*p* = 0.012) and depression (*p* = 0.043) levels decreased significantly as indicated by reduced SAS and SDS scores at the beginning and the end of therapy. However, no hypotheses confirmation on the effectiveness of LDT could be found. There was no significant nightmare reduction, comparing the nightmare frequency at initiation to end of therapy. Ratings of the interference of nightmares on quality of life and daily functioning did not change. Moreover, no changes in PTSD-profile were found. Sleep quality, daytime sleepiness, and severity of symptoms did not improve significantly. There were no significant differences regarding the parameters listed in Table 1 between the LDT group and controls.

## Discussion

Our findings show that anxiety and depression levels significantly decreased during treatment. All other parameters such as the interference of nightmares on daily functioning, and on quality of life (MQLI) in general, the severity of trauma (IE-S), daytime sleepiness, and sleep quality did not show any significant changes. These results are not surprising considering that the course of PTSD is complex and long lasting and 3 months (the time from baseline to follow-up) of treatment might be too short to cause any positive group effects. Our findings provide some support on the effectiveness of LDT in the treatment of nightmares, especially when it comes to patients with psychological disorders.

### Limitations

In this study, there was a high drop-out rate and many of the returned questionnaires were incomplete and could not be included in the statistical analyses. Therefore, sample size was quite small. Although subjects were continuously motivated to participate in the group therapy sessions, they found it difficult under their strong medication to maintain concentration which is required for LD. Although two participants reported lucid dreams and being capable of changing the dream’s content while asleep, for most subjects, LD remained novel. Because of this, conclusions drawn from differences between LDT group and controls must be assessed carefully. Although one of the inclusion criteria was “to have at least several nightmares per week,” most participants reported only several nightmares per month on average at baseline. The short treatment period might pose another relevant methodological issue which limits the ability to draw conclusions.

Aside the numerous omissions in the data set, also a high inhomogeneity within the sample was observed, even though recruitment of subjects, distribution and collection of the questionnaires were controlled. The majority of the subjects had a comorbid substance use disorder which could have had an influence on the reliability of the completion of the questionnaires as well. Low levels of resilience among subjects could be observed when offering them a polysomnographic screening in a sleep laboratory of which only two participants made use of.

All of the above indicate that single case studies should be applied and further qualitative analyses are needed.

### Future Perspectives

As positive effects of LDT on nightmares could be obtained previously (Spoormaker and van den Bout, 2006; Holzinger et al., 2015) and it is reasonable to believe that part of this study’s results are not significant due to methodological shortcomings, it is necessary to further research positive effects of LDT. With nightmares being present as symptoms in various psychological disorders, such as eating disorders, schizophrenia, depression, personality disorders, borderline disorder, substance use disorder (esp. alcohol; Fiss, 1980), and diverse organic diseases (Holzinger and Stefani, 2020), LDT is worth investigating, for it can provide a helpful tool for the ones affected. To address this, Gavie and Revonsuo (2010) propose more intense lucidity interventions over longer time periods and with larger sample sizes. Despite of the limitation due to the small sample size, the study has its strength in a naturalistic design and hot spot recruitment, reporting real word data in order so reach higher external validity and some generalizability. Therefore, the decrease in symptoms (anxiety or depression) is interesting. It has to be taken into account that LDT as an intervention technique belongs to the factors or variables that in general explain 10–15% of the variance of the therapy outcome. Taking the whole situation into consideration, other factors may also contribute to the outcome: common factors (e.g., therapeutic relationship and alliance) explaining 30%, patient variables explaining 40% of the variance, and the therapist variable explaining about 20% of the variance in therapy outcome (Lambert, 2013). Concerning further designs naturalistic studies, as well as randomized controlled trials (RCTs) should be conducted as they explain different amounts of variance, e.g., for the therapist variable the overall variance ranges from 5% in controlled studies to 17% in naturalistic studies. In this current investigation, the variables therapeutic alliance (group therapy) and patient factors (e.g., substance use disorder and dependency) might also considered having a possible influence on the symptom reduction of anxiety and depression. Further investigations are needed to investigate these moderators and mediators.

## Data Availability Statement

The datasets generated for this study will not be made publicly available in order to maintain confidentiality on behalf of the patients and their sensitive data.

## Ethics Statement

Ethical review and approval was not required for the study on human participants in accordance with the local legislation and institutional requirements. The patients/participants provided their written informed consent to participate in this study.

## Author Contributions

BH funding acquisition; BH and GK: conceptualization and methodology; data aquisition and data management; BH, GK, and BS: data analysis and first draft of the result section; BH, GK, and BS original draft preparation and finalization of the article. All authors contributed to the article and approved the submitted version.

### Conflict of Interest

The authors declare that the research was conducted in the absence of any commercial or financial relationships that could be construed as a potential conflict of interest.
